# Enhancing catechins, antioxidant and sirtuin 1 enzyme stimulation activities in green tea extract through pulse electric field-assisted water extraction: Optimization by response surface methodology approach

**DOI:** 10.1016/j.heliyon.2024.e36479

**Published:** 2024-08-17

**Authors:** Nuttinee Salee, Srisuwan Naruenartwongsakul, Wantida Chaiyana, Artit Yawootti, Kanyarat Suthapakti, Piyawan Simapaisarn, Worrapob Chaisan, Niramon Utama-ang

**Affiliations:** a Division of Product Development Technology, Faculty of Agro Industry, Chiang Mai University, Thailand; b Division of Food Engineering Development Technology, Faculty of Agro-Industry, Chiang Mai University, Thailand; c Department of Pharmaceutical Science, Faculty of Pharmacy, Chiang Mai University, Thailand; d Department of Electrical Engineering, Faculty of Engineering, Rajamangala University of Technology Lanna, Chiang Mai, Thailand; e Cluster of High Value Products from Thai Rice and Plants for Health, Chiang Mai University, Thailand

**Keywords:** Green tea, Pulse electric field, Water extraction, Catechin, Antioxidant activity, Sirtuin 1 enzyme

## Abstract

Green tea is an economic resource in Thailand because it is derived from smallholder agriculture and has expanded into food production. The purpose of this study is to optimize the parameters of pulsed electric field (PEF) assisted green tea extraction to produce a natural health product. A central composite design was involved to determine the effect of independent variables, including the intensity of electric field (I; 3–5 kV/cm), number of pulses (Np; 1000 to 3000 pulses) and green tea-to-water ratio (GT/W; 0.05–0.15 g/mL) on catechin (C), epicatechin (EC), epicatechin gallate (ECG), epigallocatechin (EGC) and epigallocatechin gallate (EGCG), total phenolic compound, antioxidant and sirtuin 1 enzyme stimulating activities. The results indicated that the Np had the most significant impact (p < 0.05) on the content of catechin and its derivatives and sirtuin 1 enzyme stimulating activity. The observations revealed that the I had a greater impact on antioxidant activities compared to the Np. The optimal conditions for PEF using the response surface method were determined to be I of 5 kV/cm, Np of 3000 pulses, GT/W of 0.14 g/mL and specific energy of 27 kJ/kg. Under the optimized conditions, the content of C, EC, ECG, EGC and EGCG were 7.34 ± 0.33, 11.26 ± 0.25, 3.75 ± 0.13, 7.53 ± 0.77 and 37.78 ± 0.58 mg/g extract, respectively. Furthermore, it was observed that green tea extract exhibited the ability to modulate the deacetylation activity of the sirtuin 1 enzyme, with a value of 22.63 ± 0.17 FIR. The results emphasized that the PEF led to achieving better responses compared to without pre-treatment using the PEF. Therefore, innovative technologies as PEF can be utilized for green tea extraction to produce natural ingredients, which can contribute to improved accessibility to healthcare. Additionally, the implementation of innovation techniques, such as PEF, in the extraction industry can enhance productivity growth and economic development.

## Graphical abstract

**Figure:**
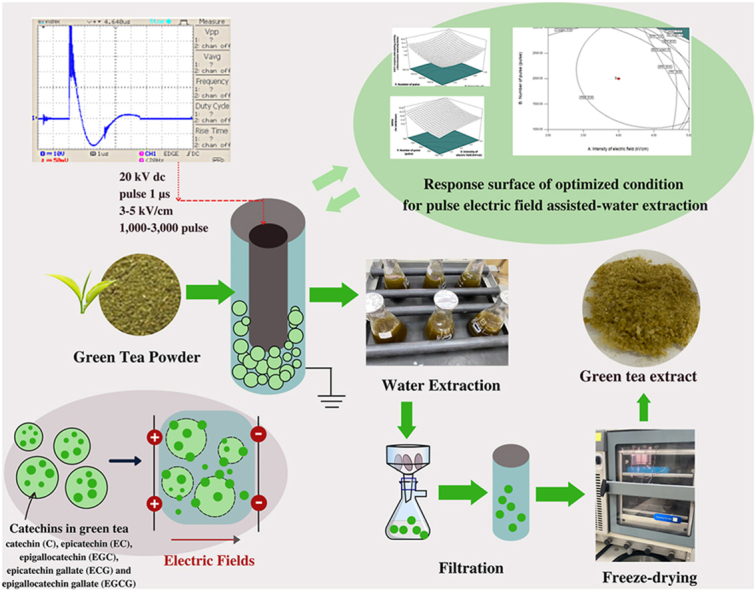

## 1 Introduction

Thailand's suitability for tea cultivation, which provides support to tea farmers and producers, has led to its international recognition as a prominent supplier of tea leaves within the global market. However, Thailand still faces challenges in terms of integrating tea processing, such as the introduction of innovative products that cater to different market applications [1]. The chemical composition of green tea is complex: proteins (15–20 % dry weight); amino acids (1–4% dry weight); carbohydrates (5–7% dry weight) such as cellulose, pectin, glucose, fructose and sucrose; minerals and trace elements (5 % dry weight); and trace amounts of lipids (linoleic and α-linolenic acids). Moreover, green tea contains polyphenols, which include flavanols, flavonoids and phenolic acids. These compounds may account for up to 30 % of the dry weight. Green tea contains a variety of catechins (flavan-3-ol) belonging to the group of flavonoids, including epigallocatechin-3-gallate (EGCG), epicatechin-3-gallate (ECG), epigallocatechin (EGC) and epicatechin (EC) [2,3]. Among the catechins found in green tea, EGCG is the most plentiful, representing approximately 60 % of the total catechin concentration, followed by EGC (approximately 20 %), ECG (approximately 14 %) and EC (approximately 6 %) [4]. The number and positions of the hydroxyl groups on the rings of green tea catechins are the structural characteristics which significantly influence their biological matter through hydrogen bonding or electron and hydrogen transfer processes [5].

Green tea catechins can decrease the generation of free radicals in the body, preventing cellular and molecular damage by chelating transition metals and scavenging excessive reactive oxygen species [6]. It is interesting that green tea catechins have the ability to produce metabolically beneficial effects by promoting the expression and activity of sirtuin 1 [7]. Sirtuin 1 is a well-known and intensively researched member of the mammalian class III histone deacetylases known to play a significant role in aging and lifespan [8]. In cellular, animal and human research, green tea and its primary component EGCG have demonstrated anti-inflammatory benefits. The anti-inflammatory effect is achieved through the suppression of nuclear factor-kappa B (NF-κB) activation [9], resulting in a decrease in the expression of inflammatory cytokines and inflammation-mediated enzymes such as TNF-α [7] and COX-2 [10]. Thus, green tea and its catechin constituents have many potentials in regard to anti-aging which has promoted the expression and activity of sirtuin 1, strong anti-inflammatory and anti-oxidant properties.

As a result of the above-mentioned health benefits attributed to green tea, researchers have subsequently devised extraction techniques aimed at maximizing the extraction of green tea catechins while maintaining their effectiveness. Traditional green tea extraction methods, such as maceration and continuous hot extraction (Soxhlet), are common and widely used. However, these techniques are less effective in terms of extraction time, residue amount, toxicity, and ability deactivation of green tea polyphenols [11,12]. While microwave and ultrasonic methods are commonly used for extracting green tea catechins, the increasing of temperatures may lead to the degradation and epimerization of catechin groups [13]. Especially in regard to the major bioactive polyphenols, as EGCG were found with degradation below 44 °C and the epimerization from gallocatechin gallate (GCG) to EGCG when exceed 98 °C [13,14]. In particular, it is known that the application of PEF is being applied as a non-thermal emerging technology for improving the extraction efficiency of bioactive compounds from varies plants, such as beetroot [15], black rice grain [16], onion [17] and tropical almond red leaves [18]. The short time pulsed with high voltage into the food product placed between two electrodes leads to membrane permeability and the increase of the extraction yield [18]. PEF is possible to extract expected intracellular components from green tea, like EGCG and other catechins, by shortening the treatment duration without requiring high temperatures and using water as a solvent. Interestingly, life cycle analysis demonstrated that CO_2_ emissions during green tea extraction generated by PEF were lower than microwaves and ultrasound [19]. However, to date, there is few research that has employed PEF as an extraction technique to augment the extraction of catechins, leading to increased antioxidant activities and anti-inflammatory properties in terms of stimulating activities of the sirtuin 1 enzyme.

Therefore, the goal of this research was to optimize the PEF parameters including intensity of electric field, number of pulses, and the ratio of water and green tea with constant pulse duration and pulse frequency for extraction. The research proposed to decide the most suitable PEF treatment to obtain extract with high catechin and catechin derivatives contents and antioxidant activities. In particular, the increased concentrations of extracted compounds from green tea may have the potential to enhance the stimulating activities of the sirtuin 1 enzyme. Furthermore, the efficacy of electric field on green tea was demonstrated by comparing the pretreatment of water extraction using PEF and non-PEF methods. This was substantiated through the analysis of surface morphology using scanning electron microscopy and Fourier transform infrared spectroscopy.

## 2 Martials and method

### 2.1 Materials and chemicals

Green tea leaves (*Camellia sinensis* var. *sinensis* cv. Chin Shin Oolong No.17) were harvested in 2018 at Chiang Rai province, Thailand. Samples were dried using tray dryer (70 °C 12 h), and then milled and sieved through a 60-mesh filter. Dried green tea powder was stored in a zip-lock bag and kept at −20 °C prior to extraction. 2,2′-diphenyl-1-picrylhydrazyl-hydrate (DPPH), 2,2′-azinobis 3-ethylbenzothiazoline-6-sulphonate (ABTS) and Folin–Ciocalteu reagent, 2,4,6 tripyridyl-s-triazine (TPTZ) were purchased from Sigma-Aldrich (St. Louis, MO, USA). Potassium persulfate (K_2_S_2_O_8_), ferrous sulfate (FeSO_4_), hydrochloric acid (HCl), sodium carbonate (Na_2_CO_3_), sodium acetate (CH_3_COONa) and acetic acid (CH_3_COOH) were purchased from RCI Labscan Co., Ltd. (Bangkok, Thailand). AR grade ethanol and HPLC grade acetonitrile were purchased from Merck (Darmstadt, Germany). Catechin (C), epicatechin (EC), epicatechin gallate (ECG), epigallocatechin (EGC) and epigallocatechin gallate (EGCG) were purchased from S.M Chemical Supplies Co., Ltd. Sirtuin 1 activity Assay Kit (Fluorometric) ab156065 was purchased from Abcam, USA.

### 2.2 Overall experimental design

Green tea powder was prepared and utilized for carrying out experiments during 2019–2020. They were placed into the PEF chamber using different ratios of green tea powder to water (GT/W). The PEF parameters, including electric field intensity and number of pulses, were adjusted using a central composite design to optimize them further using response surface methodology. Following the PEF treatment, the mixed solution underwent an additional extraction process using water for a period of 6 h at room temperature (28.5 ± 1.5 °C). After that, the mixed solution was filtered and freeze dried. The extracted green tea was analyzed for the content of catechin and its derivatives, the antioxidant activities, and stimulating activity of the sirtuin 1 enzyme. The PEF extraction process was optimized to obtain a high level of all targeted responses in the green tea extract. Additionally, a comparison was conducted between PEF and non-PEF pretreated extraction method.

### 2.3 PEF-assisted water extraction methods

The PEF machine was designed and developed by the department of Electrical Engineering, faculty of Engineering, Rajamangala University of Technology Lanna, Chiang Mai, Thailand. The electric field generator was operated by 220 VAC, 50 Hz and 500 W. The rotating gap switches generated a high voltage electric field (0–10 kV/cm and 1μs) to the chamber. The volume of the coaxial-cylinder PEF chamber is 0.57 L. In this study, the constant pulse duration and pulse repetition frequency was fixed at 1 μs and 10 Hz. The electric field strength (I; 3–5 kV/cm) and number of pulses (Np; 1000–3000 pulses) were varied to optimize the PEF-assisted extraction process. The specific energy (kJ/kg) supplied to the treated sample in the chamber was adjusted by varying the number of pulses, ranging from 3.24 to 27 kJ/kg. The specific energy intake $W_{spec,PEF}$ (kJ/kg) was determined according to the previous research [20,21], and calculated using equations (1), (2):

(1) $$W_{spec,PEF}=\frac{U^{2}CN_{p}}{2m}$$

(2) $$I=\frac{U}{d}$$

where W_spec,PEF_ is specific energy (kJ/kg), C is the capacitance (0.09 μF), Np is number of pulses and m is the mass of the treated samples (kg), I is electric field strengths (kV/cm), U is the voltage (V) and d is the distance between the electrodes (2 cm).

Green tea powder and water were loaded in the chamber for pretreatment according to conditions. 0.5 kg of a mixture of green tea powder and water was loaded in the chamber with ratios varying from 0.05 to 0.15 g/mL. The mixtures were then extracted by electrical shaker (Unimax2010, Heidolph) for 6 h. The mixtures were centrifuged, and supernatants were filtered through filter paper (Whatman® No.1, Merck, Germany) using vacuum suction filter. The collected samples were freeze-dried using a tray freeze dryer. Pressure (0.09 mBar), primary drying condition (30 °C for 8h) and secondary drying condition (−10 °C for 8h) were taken. The samples were then stored in a refrigerator at 4 °C until they were ready for use.

### 2.4 Analytical analysis of green tea extract

The freeze-dried extract was mixed with distilled water at a stock concentration of 1 mg/mL. Subsequently, the mixture was filtered through filter paper (Whatman® No.1, Merck, Germany) to prepare the sample prior to further analysis.

#### 2.4.1 Determination of catechin and catechin derivatives content

Catechin and catechin derivatives were determined according to the high performance liquid chromatography (HPLC) method of Sirisa-Ard, P. et al. (2017) [22] and were performed on Agilent HPLC 1200 Series (Agilent Technologies, Santa Clara, CA, USA), which was controlled by ChemStation software. The column chromatography was C_18_ reversed phase (4.6 x 250 mm. Waters, Ireland). The mobile phases consisted of the 86.5 % v/v phosphoric acid (0.2 % v/v) in 12 % acetonitrile and 1.5 % v/v tetrahydrofuran (mobile phases A) and 73.5 % v/v phosphoric acid (0.2 % v/v) in 25 % acetonitrile and 1.5 % v/v tetrahydrofuran (mobile phases B) in a gradient mode. The linear gradient of elution was followed: 30 min, 100 % of A; 0–30 min, 10–100 % of B; 30 min, 10–100 % of A; 30 min 100 % of A with the flow rate 1 mL/min. The wavelengths of the UV detector were settled at 210 nm and 280 nm with controlled column temperature at 25 °C. The results were compared with the peak area of standards to determine the content of catechin and catechin derivatives in the green tea extract.

#### 2.4.2 Determination of total phenolic compounds (TPC)

TPC of green tea extract was determined by the Folin–Ciocalteu colorimetric method described by Chaiyana et al., with some modifications [23]. Concisely, Folin-Ciocalteu reagent (diluted 1:10) was mixed with the extract solution (0.01 mg/mL) in a 96-well polypropylene microplates. The mixed solutions were incubated at room temperature in the dark for 4 min. Then, 7.5 % (w/v) Na_2_CO_3_ was added to the mixture and incubated at room temperature for 2 h. A microplate reader (DTX880, Beckman Coulter, Austria) was used for measuring the absorbance of the mixtures at 760 nm. The standard curve of gallic acid was drawn.

#### 2.4.3 Determination of antioxidant activities

(1) DPPH Assay

Scavenging activity against DPPH radicals was determined using the method described by Chaiyana et al., with some modifications [23]. Briefly, 180 μL of the 167 μM of DPPH solution was mixed with 20 μL of 0.001 mg/mL extract solution and incubated in the dark for 30 min. The absorbance of the mixture was measured at 520 nm using a microplate reader. The scavenging activities on the DPPH of the extract were reported as % inhibition which was calculated using equation (3):

(3) $$\%\;\text{DPPH}\;\text{scavenging}\;\text{activity}=\left(\frac{C-S}{C}\right)x\;100\%$$

where C was UV absorbance of control solution and S was UV absorbance of the sample solution.

(2) ABTS Assay

Scavenging activity against ABTS radicals was determined using the method described by Chaiyana et al., with some modifications [23]. Briefly, 3 mL of 2.45 mM K_2_S_2_O_8_ solution was mixed with 2 mL of 7 mM ABTS solution and incubated in the dark for 24 h. Then 180 μL of ABTS solution was added to 20 μL of 0.02 mg/mL extract solution in a 96-well plate and incubated at room temperature for 5 min. The UV absorbance of the mixture was measured at 750 nm using a microplate reader. The standard curve of Trolox was drawn.

(3) FRAP Assay

Ferric reducing power was determined using the method described by Chaiyana et al., with some modifications [23]. FRAP solution was prepared freshly by mixing 10 mM 2,4,6-TPTZ solution in 40 mM HCl (1 mL) with 20 mM ferric chloride (1 mL) and 0.3 M acetate buffer pH 3.6 (10 mL). For analysis, 20 μL of 0.1 mg/mL extract solution was mixed with 180 μL of FRAP solution in a 96-well plate. The mixture was vortexed and then left to stand in dark place for 5 min. The UV absorbance of the mixture was measured at 595 nm. The standard curve of FeSO_4_ was drawn.

#### 2.4.4 Determination of sirtuin 1 enzyme-stimulating activity

Quantification of sirtuin 1 enzyme-stimulating activity was determined using the Abcam's Sirtuin 1 Activity Assay Kit (Fluorometric, ab156065) according to the manufacturer's protocol. Briefly, 25 μL of H_2_O (HPLC grade), 5 μL of sirtuin 1 assay buffer, 5 μL of fluoro-substrate peptide and 5 μL of NAD were mixed in microplate wells. Then 5 μL of developer was added to each well of the microplate wells. The initiate reaction was started by adding 5 μL of sample to each well and mixing thoroughly at room temperature. The fluorescence intensity was measured at 2-min intervals for a duration of 60 min using microplate fluorimeters (SpectraMax M3, Molecular Devices, USA) with an excitation wavelength of 360 nm and an emission wavelength of 485 nm. The stimulating activity of the extract on the sirtuin 1 enzyme was calculated by determining the ratio of fluorescent intensity between the samples and the control in the unit of fluorescence intensity ratio (FIR) [24].

### 2.5 Scanning electron microscopy (SEM)

Green tea powder before and after pretreatment by PEF were manually snapped on the surface of the stub and coated with gold (Au). The samples were observed by SEM (Prisma E− 51-ADD0130, Thermo Fisher Scientific, USA) under an acceleration voltage of 10 kV, at a magnification of 800x and 3,000x.

### 2.6 Fourier transform infrared (FT-IR) spectroscopy analysis

FT-IR analysis was conducted to identify the functional groups of the active components present in green tea powder. Dried green tea powder (2 mg) was added to the sample chamber. The green tea powder obtained through PEF treatment (2 mg) was quickly blotted with paper towels to remove residual water on the surface, and then added to the sample chamber. The FT-IR spectrum of samples in the wavenumber range of 4000 to 500 cm^−1^ with a resolution 4 cm^−1^ was obtained by FT-IR Spectrometer (FT/IR-4700, Jasco International Co. Ltd., Tokyo, Japan).

### 2.7 Optimization of PEF-assisted extraction

The optimization analysis of the green tea extraction process purposes at determining the optimal combination of various PEF extraction parameters for enhanced catechin and catechin derivatives content, TPC, antioxidant activities and sirtuin 1 enzyme-stimulating activity. The experimental design and analysis of data was performed by using Design Expert 6.0.10 (Stat-Ease, Inc., Minneapolis, MN) software. Central Composite Design (CCD) with α = ±1.68 was chosen for applying to obtain the optimal conditions for PEF of green tea. The independent parameters were the intensity of the electric field (I, ranged from 3 to 5 kV/cm), number of pulses (Np, ranged from 1000 to 3000 pulses) and the ratio of green tea powder to water (GT/W ranged from 0.05 to 0.15 g/mL). Meanwhile TPC (mg GAE/g), DPPH (%inhibition), ABTS (mol Trolox/g extract), FRAP (mmol FeSO_4_/g), sirtuin 1 enzyme-stimulating activity (FIR), catechin and catechin derivatives (mg/g extract) were selected as responses. A set of 17 experiments was performed to study and the central point was repeated three times.

Mathematical models between the independent variables were evaluated by means of multiple linear regression analysis of equation (4) [25].

(4) $$Y=\beta_{0}+\sum_{i=1}^{n}\beta_{i}X_{i}+\sum_{i=1}^{n}\beta_{ii}X_{i}^{2}+\sum_{i=1}^{n}\sum_{j=i+1}^{n-1}\beta_{ij}X_{i}X_{j}+e$$

where $\beta_{0}$, $\beta_{i}$, $\beta_{ii}$, $\beta_{ij}$ are constant coefficients of regression, $X_{i}$ and $X_{j}$ are the independent variables, Y is the dependent variables, n is number of independent variable and e is the random error term.

Analysis of Variance (ANOVA) was performed to obtain the coefficients of the accuracy equation. All the variables of polynomial regression at a significance level of p < 0.05 were included in the model and the coefficient of determination (R^2^) was generated to assess the adequacy of the model. The response surfaces were generated from the equations of the second order polynomial, using the values of each independent variable giving to the maximum quadratic response [26].

## 3 Results and discussion

### 3.1 Effect of PEF process variables on response values

The effects of three process variables (intensity of electric field, number of pulses and ratio of green tea to water) on response values are presented in Table 1.

**Table 1:** The experimental design involved applying PEF to green tea extraction and the observed responses.

Run	Factor			Responses^a^									
				Content of catechins (mg/g of extract)					TPC (mg GAE/g)	Antioxidant Activities			Sirtuin 1- activity (FIR)
	I (kV/cm)	Np (pulse)	GT/W (g/mL)	C	EC	ECG	EGC	EGCG		DPPH (% inhibition)	ABTS (mol Trolox/g)	FRAP (mmol FeSO_4/_g)	
1	3 (−1)	1000 (−1)	0.15 (+1)	5.35 ±0.01	8.03 ±0.03	3.08 ±0.01	6.41 ±0.01	29.61 ±0.02	169.87 ± 5.77	38.52 ± 0.50	31.51 ± 0.23	7.72 ± 0.04	7.37 ± 0.04
2	3 (−1)	1000 (−1)	0.05 (−1)	3.82 ±0.07	4.88 ±0.04	2.92 ±0.01	8.42 ±0.52	23.53 ±0.02	70.37 ± 0.64	31.23 ± 0.15	30.79 ± 0.04	7.22 ± 0.38	8.55 ± 0.14
3	3 (−1)	3000 (+1)	0.15 (+1)	7.60 ±0.11	10.76 ±0.09	3.23 ±0.12	8.28 ±0.52	40.41 ±0.06	138.33 ± 1.96	44.89 ± 0.83	35.69 ± 3.44	17.66 ± 0.76	18.00 ± 0.09
4	3 (−1)	3000 (+1)	0.05 (−1)	7.86 ±0.04	11.73 ±0.01	3.28 ±0.03	6.51 ±0.44	28.05 ±0.01	447.63 ± 1.84	40.48 ± 0.67	29.50 ± 0.54	15.77 ± 0.42	12.14 ± 0.01
5	4 (0)	3681 (+α**)**	0.10 (0)	8.62 ±0.11	13.22 ±0.10	3.36 ±0.01	9.74 ±0.74	31.23 ±0.05	380.09 ± 1.06	40.46 ± 0.92	48.34 ± 0.09	27.11 ± 0.42	20.10 ± 0.76
6	4 (0)	318 (-α**)**	0.10 (0)	4.86 ±0.11	6.75 ±0.07	3.02 ±0.08	6.92 ±0.57	14.76 ±0.05	330.96 ± 2.13	29.05 ± 0.39	17.29 ± 0.38	9.71 ± 1.17	2.86 ± 032
7	4 (0)	2000 (0)	0.18 (+α**)**	6.88 ±0.10	8.22 ±0.07	3.28 ±0.04	10.59 ±0.78	35.73 ±0.05	283.81 ± 1.82	48.45 ± 0.60	34.32 ± 2.75	26.91 ± 0.60	5.74 ± 0.56
8	4 (0)	2000 (0)	0.02 (-α**)**	4.62 ±0.23	6.90 ±0.16	3.15 ±0.09	6.56 ±0.78	34.86 ±0.11	266.86 ± 1.96	45.56 ± 0.63	28.14 ± 0.22	17.51 ± 0.71	2.49 ± 0.37
9 ^b^	4 (0)	2000 (0)	0.10 (0)	6.88 ±0.12	9.23 ±0.07	3.21 ±0.04	7.29 ±1.57	36.40 ±0.05	287.98 ± 1.06	46.72 ± 0.87	41.56 ± 2.95	24.00 ± 0.71	7.83 ± 0.15
10 ^b^	4 (0)	2000 (0)	0.10 (0)	7.06 ±0.08	10.40 ±0.59	3.14 ±0.03	6.29 ±0.11	37.17 ±0.39	263.42 ± 1.84	43.53 ± 1.63	40.45 ± 1.30	24.28 ± 0.49	8.52 ± 0.13
11 ^b^	4 (0)	2000 (0)	0.10 (0)	7.00 ±0.02	9.08 ±0.23	3.10 ±0.01	6.29 ±0.11	36.30 ±0.15	232.83 ± 0.91	46.44 ± 2.67	40.58 ± 0.31	24.38 ± 0.74	9.07 ± 0.01
12	5 (+1)	1000 (−1)	0.15 (+1)	5.69 ±0.02	8.25 ±0.48	3.03 ±0.03	5.72 ±0.18	28.60 ±0.25	631.84 ± 1.84	38.99 ± 1.26	35.84 ± 0.13	18.95 ± 0.59	12.55 ± 0.21
13	5 (+1)	1000 (−1)	0.05 (−1)	5.17 ±0.04	7.05 ±0.01	3.28 ±0.01	5.89 ±0.01	34.96 ±0.01	367.81 ± 1.06	38.27 ± 1.20	29.42 ± 1.87	17.40 ± 0.14	3.53 ± 0.26
14	5 (+1)	3000 (+1)	0.15 (+1)	8.64 ±0.01	11.78 ±0.47	3.18 ±0.03	6.09 ±0.24	38.08 ±0.31	238.86 ± 1.06	48.50 ± 0.91	38.96 ± 0.40	28.29 ± 0.02	25.85 ± 0.79
15	5 (+1)	3000 (+1)	0.05 (−1)	7.02 ±0.01	9.80 ±0.04	3.31 ±0.02	6.08 ±0.01	22.06 ±0.02	306.40 ± 1.08	58.03 ± 1.47	42.98 ± 0.78	25.21 ± 0.04	8.28 ± 0.48
16	5.7 (+α**)**	2000 (0)	0.10 (0)	8.64 ±0.04	12.35 ±0.12	3.09 ±0.06	6.81 ±0.01	38.46 ±0.08	429.21 ± 1.84	47.48 ± 1.27	42.74 ± 0.96	27.86 ± 0.97	25.48 ± 0.22
17	2.3 (-α**)**	2000 (0)	0.10 (0)	6.67 ±0.13	9.17 ±0.46	3.14 ±0.03	7.17 ±0.01	36.36 ±0.30	128.95 ± 1.12	36.32 ± 0.94	27.62 ± 0.73	9.55 ± 0.02	12.10 ± 1.26

Note: ^a^ Analytical results are the average of triplicates (mean ± SD). And ^b^ three runs of center point.

I = Intensity of electric field (kV/cm), Np = Number of pulse (pulses), GT/W = Ratio of green tea and water (g/mL).

#### 3.1.1 Catechin and catechin derivatives contents

The content of catechin and catechin derivatives in green tea extracts obtained from PEF on CCD are listed in Table 1. The content of C, EC, ECG, EGC and EGCG of extract ranged from 3.82 ± 0.07 to 8.64 ± 0.04, 4.88 ± 0.04 to 12.35 ± 0.12, 2.92 ± 0.01 to 3.36 ± 0.01, 5.72 ± 0.18 to 9.74 ± 0.74 and 14.76 ± 0.05 to 40.41 ± 0.06 mg/g extract, respectively. The highest electric field intensity of 5.7 kV/cm with number of pulse 2000 pulses and 0.10 g/mL of green tea/water (run 16) yielded the highest catechin content (8.64 ± 0.04 mg/g), however the result was similar to those obtained with the highest number of pulses, 3861 pulses with electric field-intensity 4 kV/cm (run 5). Furthermore, the highest values of EC, ECG and EGC were obtained by PEF under conditions of run 5. Conversely, the highest value of EGCG was found with the highest electric field intensity of 3 kV/cm with number of pulse 3000 pulses and 0.15 g/mL (run 3). The results indicated that varying levels of the process variables had distinct effects on the catechin and catechin derivatives obtained from the green tea extract. This funding was consistent with the research conducted by Kandušer et al. [27], which demonstrated that the parameters of PEF, such as the intensity of the electric field, pulse characteristics and number of pulses had an impact on the extraction of natural compounds from the plant matrix.

#### 3.1.2 Total phenolic compounds and antioxidant activities

The total phenolic compounds and antioxidant activities (DPPH, ABTS and FRAP) of green tea extract obtained from PEF on CCD are listed in Table 1. TPC values of green tea extract varied from 70.37 ± 0.64 to 631.84 ± 1.84 mg GAE/g extract. The lowest TPC of the extract was obtained when the electric field intensity was 3 kV/cm and the ratio of green tea to water was 0.05 g/mL (run 2). However, the highest TPC of the extract was obtained when the electric field intensity was 5 kV/cm and the ratio of green tea to water was 0.15 g/mL (run 12) with the same number of pulses (1000 pulses). The results indicated that increasing the electric field intensity along with a higher ratio of green tea to water could enhance the TPC of the obtained extract while maintaining a constant number of pulses.

Furthermore, the findings could provide support for the studies conducted by Redondo, D. et al. and Salee, N. et al. which explained that electric field within the range of relatively low strengths (3–5 kV/cm) was found to be effective in extracting bioactive compounds from plants. This was attributed to the induction of electroporation in the cell wall, resulting in increased permeability for the transportation of macromolecules. Moreover, it facilitated the extraction of bioactive compounds from the cells thereby enhancing the extraction yield [16,28].

The antioxidant activities were obtained by DPPH, ABTS and FRAP assay which their resulting values changed between DPPH (%inhibition) ranged from 29.05 ± 0.39 to 58.03 ± 1.47, ABTS (mol Trolox/g extract) ranged from 17.29 ± 0.38 to 48.34 ± 0.09 and FRAP (mmol FeSO4/g) ranged from 7.22 ± 0.38 to 28.29 ± 0.02. The highest DPPH and FRAP values of extract were extracted from electric field intensity 5 kV/cm and number of pulses was 3000 pulses (run 15 and run 14, respectively), while the highest ABTS value was extracted from electric field intensity 4 kV/cm and number of pulses was 3681 pulses (run 5). The results indicated that a high intensity of electric field and a greater number of pulses or treatment time had a positive effect on antioxidant potential, while the ratio of green tea to water was also taken into consideration. However, it was observed that a slight decrease in antioxidant activities occurred when the electric field was increased to 5.7 kV/cm, which was similar to the report that when the electric field was increased to 5 kV/cm, the total phenolic content and antioxidant activities of blueberries slightly decreased following a pre-treatment with PEF [29]. This result related the research of Mahnič-Kalamiza, S. E. et al. which demonstrated that high levels of electrical energy delivered during PEF treatment may lead to a decrease in antioxidant activities, due to the deterioration of bioactive compounds [30].

#### 3.1.3 Sirtuin 1- activity

Table 1 shows that the sirtuin 1-activity of green tea extract ranged from 2.49 ± 0.37 to 25.85 ± 0.79 units of FIR. The results suggested that the highest sirtuin 1-activity of the extract (25.85 ± 0.79) was obtained when the electric field intensity was 5 kV/cm, the number of pulses was 3,000, and the ratio of green tea to water was 0.15 g/mL. This value was very close when the electric field intensity was 5.7 kV/cm, the number of pulses was 2,000, and the ratio of green tea to water was 0.10 g/mL (25.48 ± 0.22). Thus, when the electric field intensity was increased from 5 kV/cm to 5.7 kV/cm, along with a decrease in the number of pulses and the ratio of green tea to water, the observed sirtuin 1-activitiy values were found to be very similar. Additionally, when the electric field intensity was maintained at a constant value of 5 kV/cm and the ratio of green tea to water was 0.15 g/mL, increasing the number of pulses from 1000 to 3000 resulted in an increase of sirtuin 1-activity. The activities increased from 12.55 ± 0.21 (run 12) to 25.85 ± 0.79 (run 14), representing an approximate twofold increase.

It was discovered that three input variables influenced the responses of the pulse electric field extraction process, however, it was challenging to choose the appropriate condition or run of experiment in Table 1. Consequently, to attain high catechin and catechin derivatives contents as well as antioxidant and sirtuin 1 enzyme-stimulating activities, optimization using response surface modeling (RSM) was employed in the subsequent data analysis in Table 2.

**Table 2:** The regression equations derived based on the ANOVA analysis of the PEF process applied to green tea extraction.

Model Variables	C		EC		ECG		EGCG		TPC		DPPH		ABTS		FRAP		Sirtuin 1- activity	
	Coefficient	p-value	Coefficient	p-value	Coefficient	p-value	Coefficient	p-value	Coefficient	p-value	Coefficient	p-value	Coefficient	p-value	Coefficient	p-value	Coefficient	p-value
β	−1.399		0.994		2.928		−10.068		−656.526		−18.855		8.424		−61.431		78.340	
I	1.032	0.007	−1.594	0.101	0.015	0.538	6.759	0.711	154.902	<0.0001	13.059	0.008	3.305	0.040	24.619	<0.0001	−34.352	0.182
Np	0.127	0.001	0.420	0.002	0.911	0.002	0.025	0.030	0.458	0.806	0.015	0.001	0.525	0.003	0.015	<0.0001	−5.919 x 10 ^−3^	0.004
GT/W	10.546	0.128	81.585	0.074	−0.069	0.887	42.251	0.083	−564.392	0.885	216.448	0.391	28.849	0.338	93.033	0.021	−126.995	0.037
I^2^			0.333	0.290			0.066	0.956	4.144	0.629	−0.983	0.192			−2.443	0.005	3.733	0.009
Np^2^			0.592	0.844			−0.050	0.003	0.311	0.006	−0.350	0.001			−0.254	0.004	0.115	0.092
(GT/W)^2^			−319.380	0.029			−272.354	0.582	1128.051	0.741	328.796	0.267			−482.436	0.091	−581.374	0.316
I*Np			−4.129 x 10 ^−4^	0.271			−0.234	0.138	−0.100	<0.0001	0.171	0.073			−0.169	0.824	0.477	0.580
I*(GT/W)			2.495	0.729			−21.956	0.458	1015.722	0.001	−51.318	0.016			5.572	0.715	54.792	0.020
Np*(GT/W)			−8.299 x 10 ^−3^	0.269			0.071	0.038	−1.851	<0.0001	−0.033	0.082			0.729	0.634	0.038	0.125
p-value of the Model		0.0014^a^	0.0048^a^		0.0169^a^		0.0276^a^		<0.0001^a^		0.0007^a^		0.0066^a^		0.0003^a^		0.0060^a^	
Lack of fit		0.0054^a^	0.3429 ^ns^		0.2988 ^ns^		0.0103^a^		0.5960 ^ns^		0.3726 ^ns^		0.0107^a^		0.0064^a^		0.0260^a^	
*R*^*2*^		0.6851	0.9172		0.5315		0.8565		0.9820		0.9533		0.5972		0.9655		0.9116	
Adj. *R*^*2*^		0.6124	0.8106		0.4234		0.6721		0.9587		0.8933		0.5042		0.9211		0.7979	
Pred. *R*^*2*^		0.4237	0.3810		0.1337		−0.1539		0.8865		0.6746		0.3231		0.7391		0.3321	
Adeq. Precision		9.761	10.090		7.103		8.277		26.745		15.739		7.687		14.754		10.583	

Note: β_0_ = Intercept, I = Intensity of electric field (kV/cm), Np = Number of pulse (pulse), GT/W = Ratio of green tea and water (g/mL).

^a^ significant *(p* < 0.05), ^ns^ not significant *(p* > 0.05).

### 3.2 Model fitting of PEF parameters on responses

The selected model was evaluated by calculating the coefficient of determination (R^2^ value) and multiple regression was used to analyze the correlation between independent and dependent variables [31]. The data points obtained from the responses were incorporated into a polynomial regression and are displayed in Table 2. The models of catechin and catechin derivatives content (excluding EGC), total phenolic compounds, antioxidant activities and sirtuin 1-activity were found to be statistically significant, with a p-value of less than 0.05, indicating the models' ability to effectively explore the reliable dependent variables. The models were significant at F value of 9.43 (p < 0.01) for C, 8.61 (p < 0.01) for EC, 4.92 (p < 0.05) for ECG, 6.76 (p < 0.05) for EGCG, 5.08 (p < 0.0001) for TPC, 11.64 (p < 0.01) for DPPH, 6.42 (p < 0.05) for ABTS, 8.44 (p < 0.05) for FRAP and 7.68 (p < 0.05). The lack of fit of EC, ECG, TPC and DPPH were non-significant, suggesting that they were adequately suited to the experimental design. The R^2^ value of TPC, DPPH and FRAP models were all above 0.95, indicating the goodness of the fitting model and the maximum similarity between the predicted and actual responses [31]. Although, some models (EC, ECG, EGCG, DPPH, and SIRT1) displayed a difference greater than 0.2 between the “Pred. *R*^*2*^” and “Adj. *R*^*2*^” values, which might indicate a block effect or a possible problem with our models and/or data. However, all models had “Adeq Precision” values exceeding 4, indicating an appropriate signal and could be applied for design navigation. “Adeq Precision” measures the signal to noise ratio. A ratio greater than 4 is desirable [32].

#### 3.2.1 Catechin and catechin derivatives contents

PEF process was employed to extract catechin and catechin derivatives from green tea, with the electroporation of the green tea's wall cell resulting in an increased extraction efficiency. Furthermore, the PEF can cause the disruption of vacuoles, which contain a significant amount of plant active substances and encourages the release of bioactive compounds from plants [33,34]. A statistical regression analysis was conducted to establish the correlation between electric field intensity, number of pulses and the ratio of green tea to water. A linear model was developed to predict the C and ECG, and a quadratic model was developed to predict the EG and EGCG. The electric field intensity and number of pulses in model, except the ratio of green tea to water, were having significant effect on C content (p < 0.05).

**Fig. 1:**
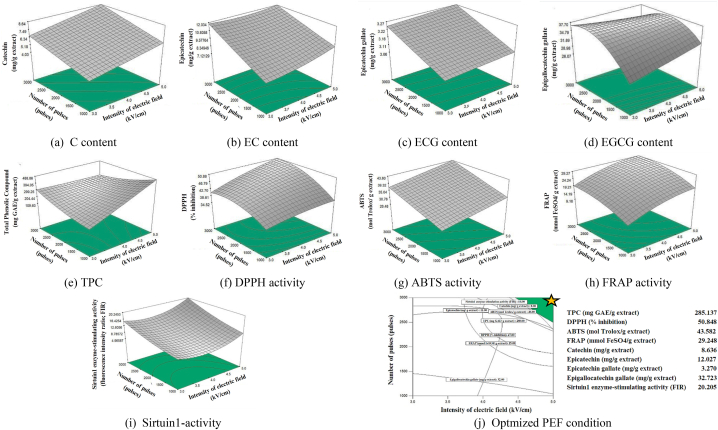
Response surface plots of response values of green tea extract as affected by electric field intensity, number of pulses and green tea to water ratio (a) C (b) EC (c) ECG (d) EGCG content (e) TPC (f) DPPH (g) ABTS (h) FRAP activities (i) Sirtuin 1- activity and (j) the optmized condition for PEF process of green tea.

Furthermore, the coefficient model of the ratio of green tea to water was the highest (10.546), indicating that the C value was significantly increased when the ratio of green tea was increased with the constant electric field intensity and number of pulses. Fig. 1(a) illustrates the surface plot of C as a function of electric field intensity and number of pulses with a constant ratio of 0.1 g/mL of green tea to water. An increase in electric field intensity and pulse number resulted in a marked increase in C. There was a significant positive correlation between the number of pulses and the contents of EC, EGC and EGCG (p < 0.05). For the EC model, when the ratio of green tea to water was squared (GT/W^2^), there was a significant decrease in the EC content of the green tea extract, as indicated by the coefficient (−319.3). The curve trend line in Fig. 1(b) illustrates that as the constant of electric field intensity and number of pulses increase, EC gradually increases. Conversely, when the constant of number of pulses and electric field intensity increases, the EC tends to decrease.

The characteristic plot of ECG against independent variables in Fig. 1(c) displays a similar trend in terms of C content. It is not in relation with previous research that reported that the various epi-structured catechins (EGCG, EGC, ECG and EC) and non-epi-structured catechins (C) had distinct characteristics when extracted using hot water. For instance, the yield of the epi-structured catechins plateaued at around 30 min, whereas the non-epi-structured catechins kept increasing with longer extraction times up to 120 min [35]. However, this study examined the effect of PEF independent variables, which could lead to different characteristics of catechin extraction using water. For the EGCG model, which is the major catechin found in green tea [36], increasing all the independent variables was effective in increasing the EGCG content. Although the higher number of pulses was more effective, the square of the number of pulses (Np^2^) had a significant effect on the decrease of EGCG (p < 0.05). This may be due to the temperature rise caused by the electric current flowing in the chamber [37] which may lead to the deterioration of the EGCG structure [38]. This pattern is demonstrated in the response surface plot of EGCG content as a function of the number of pulses and electric field intensity in Fig. 1(d). According to this result, it was clear that the number of pulses can significantly be strongly effective on extraction yield of C, EC, EGC and EGCG (p < 0.05). Additionally, the effective of catechins from PEF-assisted water extraction were contingent upon the specific energy delivered to the green tea, which can be modified by varying the electric field intensity, number of pulses and ratio of green tea to water which was in agreement with previously findings [39].

#### 3.2.2 Total phenolic compounds and antioxidant activities

Phenolic compounds were reported to be extracted at relatively low electric field strengths (0.5–5 kV/cm), potentially due to their relatively small molecular size [40,41]. This research investigated the effect of varying electric field strengths or intensities, ranging from 2.3 to 5.7 kV/cm, on the extraction of phenolic compounds from green tea. Table 2 presents the fitted quadratic model and the statistical characteristics of the model terms that were selected based on the data points from the TPC responses. The electric field intensity had a significant effect on TPC (P < 0.0001) with a high and positive coefficient of 154.902. Additionally, the interaction between electric field intensity with number of pulses and ratio of green tea to water (I*Np and I*(GT/W)) had a significant effect on TPC (p ≤ 0.001). The electric field intensity had a significant effect on TPC (p < 0.01) which was similar to the previous results which used PEF for the extraction of valuable compounds from perilla seed [31]. Fig. 1(e) shows the response surface plot of TPC as a function of electric field intensity and number of pulses. The results indicated that when the electric field intensity was increased from 3 kV/cm to 4 kV/cm, there was a gradual increase in TPC to approximately 236 GAE/g. When the electric field intensity was increased from 4 kV/cm to 5 kV/cm, there was a rapid increase in TPC to approximately 425 GAE/g. The rapid increase in TPC may be influenced by the square of the number of pulses (Np^2^) and the interaction between electric field intensity and number of pulses, as this may result in a specific energy being delivered to the green tea and an increase in the yield of phenolic compounds. This effect was consistent with the findings of other studies, which suggested that the observed phenomenon was likely caused by an increased electro-permeabilization of the cell membrane at a higher intensity of electric field [42].

The regression model and statistical coefficients of PEF parameters on scavenging activity against DPPH radicals of green tea extraction are presented in Table 2, with a response surface plot depicted in Fig. 1(f). The model showed that increasing of DPPH activity significantly affects both pulse electric field and number of pulses (p < 0.01). The response surface plot with a constant ratio of 0.1 g/mL of green tea to water indicated that the increasing intensity of electric field from 3 to 5 kV/cm resulted in a linear increase in DPPH activity, while the increasing number of pulses from 1000 to 2500 pulses led to an increase in DPPH activity that tended to stabilize when the number of pulses exceeded 2500 pulses. The increasing of DPPH activity due to PEF treatment appears to be in agreement with the findings, which investigated the extraction of phenolic compounds from fresh rosemary leaves [43].

The effect of PEF parameters on scavenging activity against ABTS radicals of green tea extract are illustrated in Table 2. The ABTS model demonstrated that a significant increase in ABTS activity had a considerable influence on both the pulse electric field and the number of pulses (p < 0.05), which is likely indicative of the DPPH activity model. Fig. 1(g) demonstrates that the augmentation of the pulse electric field from 3 kV/cm to 5 kV/cm and the number of pulses from 1000 to 3000 resulted in a linear trend of increased ABTS activity from 29.34 to 40.75 mol Trolox/g extract. Some research studies have observed that the highest capacity of the extract to neutralize the ABTS + radical by TEAC assay was obtained when an electric field strength of 2.5 kV/cm was applied via pulsed electric field (PEF) on bioactive compounds from the mushroom (*Agaricus bisporus*) [44].

The characteristics of ferric reducing power of green tea extract through PEF parameter is demonstrated in Table 2 and the response surface is plotted and illustrated in Fig. 1(h). The three main PEF parameters had significant impact on FRAP activity (p < 0.05). Furthermore, it was determined that the square terms of the electric field intensity and the number of pulses (I^2^ and Np^2^) had a significantly negative influence on FRAP activity (p < 0.01). The model indicated that the interaction terms had no effect on FRAP activity (p > 0.05). The response surface graph illustrates the change in FRAP activity when the electric field intensity and the number of pulses were increased at a constant ratio of 0.1 g/mL of green tea to water. The FRAP activity linearly increased to approximately 12 mmol FeSO_4_/g extract when the electric field intensity was less than 3.5 kV/cm and the number of pulses was less than 1,500, and then gradually increased until the electric field intensity was 4.5 kV/cm, and the number of pulses was 2500 before tending to remain constant. The decreasing trend of behavior, similar to prior studies, observed that increase in electric field intensity and the number of pulses can lead to an increase in the specific energy supplied, resulting in the degradation of sensitive compounds, which may be phenolic compounds due to a slight rise of temperature [45,46].

#### 3.2.3 Sirtuin 1-activity

Catechin group has been identified as a natural sirtuin 1 modulator, which has enabled the discovery of novel small molecules that can activate the sirtuin 1 enzyme activity [47,48]. The sirtuin 1-activity model in Table 2 demonstrates that the number of pulses and the ratio of green tea to water had a significant effect on sirtuin 1-activity (p < 0.05), while the effect was observed to have a negative trend. It was discovered that the square terms of the electric field intensity (I^2^) and the interaction term of the electric field intensity and the ratio of green tea to water (I*(GT/W)) had a significantly positive effect on the trend (p < 0.05).

Fig. 1(i) elucidates the influence of electric field intensity and pulse number on the activity of sirtuin 1 during PEF treatment of green tea extraction. At a constant electric field intensity, the number of pulses increased from 1000 to 3000 resulting in a linear increase in sirtuin 1-activity. While the effect of electric field intensity interacted with number of pulses, sirtuin 1-activity gradually decreased as electric field intensity ranged from 3.5 to 4.0 kV/cm and then slowly increased when electric field intensity exceeded 4.0 kV/cm. The activity of sirtuin 1 increased when the electric field intensity was higher than 4.0 kV/cm, which may be due to the release of other compounds in addition to phenolic compounds, such as amino acids, which may include l-Theanine. This in turn inhibits the expression of nuclear factor-κB (p65) protein by activating sirtuin 1 [49,50].

This study has demonstrated that PEF parameters have a positive influence on catechins content, total phenolic compounds, antioxidant activities and sirtuin 1-activity. Due to the varied effects of PEF parameters, response surface modeling (RSM) was employed to identify the optimal PEF extraction condition to achieve maximum responses.

### 3.3 Optimization and model validation

The model developed for the responses using PEF parameters was used to predict the optimal values of the independent variables. The results are presented in Table 3. The objective was to maximize the values of all variables, including the contents of catechin and catechin derivatives, antioxidant activities, and sirtuin 1-activity. The solution with the highest desirability was utilized to determine the optimal values of both the independent and dependent variables. From optimization results, the final desirability achieved was 77.6 %. The maximum content of C, EC, ECG and EGCG in the green tea extract was found to be 8.64, 12.03, 3.27 and 32.72 mg/g of extract, respectively. The maximum TPC was measured to be 285.14 mg GAE/g, while the DPPH was 50.85 % inhibition. The maximum ABTS was 43.58 mol Trolox/g, the FRAP was 29.25 mmol FeSO_4_/g and the sirtuin 1-activity was 20.21 FIR. These optimal values could be obtained at an electric field intensity of 5 kV, number of pulses at 3000 and a ratio of green tea to water of 0.14 g/mL. The specific energy of this optimum condition was 27 kJ/kg, which is comparable to the specific energy required for extracting polyphenols from thick and tough cell wall materials like cocoa bean shells and vanilla pods [51]. Dried green tea powder requires a higher specific energy input compared to fresh green tea leaves (22 kJ/kg), which is influenced by the inherent and electrical properties of the food being treated [52].An electric field strength of 12–45 kV/cm is usually considered sufficient for extracting beneficial compounds, specifically polyphenols, from food products [53]. Additionally, the optimal condition was achieved when the electric field had the highest range, indicating that a higher yield might potentially be obtained by increasing the electric field intensity and number of pulses. Nevertheless, in our research, applying a PEF higher than 5 kV/cm and 3000 pulses led to extraction temperatures exceeding 60 °C, which could affect the degradation of bioactive chemicals during extraction. The optimal experimental points were utilized to validate each model. The results in Table 3 were obtained in triplicate and the means were statistically compared with the predicted values provided by the models. The percentage error between the observed value and the predicted value from the optimal condition ranged from 6.41 % to 15.4 %. The observed values of ECG, EGCG and sirtuin 1 enzyme-stimulating activity were higher than the predicted values, while the remaining response values were lower than the predicted values.

**Table 3:** Validation of optimal condition of PEF process for green tea extraction.

Response variables	Optimal Condition^1^			Water Extraction^2^
	Predicted value	Observed values^3^	%Error^4^	Observed values^3^
**C** (**mg/g extract)**	8.636	7.34 ± 0.33^a^	15.07	4.66 ± 0.39^b^
**EC** (**mg/g extract)**	12.027	11.26 ± 0.25^a^	6.41	9.49 ± 0.27^b^
**ECG** (**mg/g extract)**	3.270	3.75 ± 0.13^a^	14.60	2.58 ± 0.01^b^
**EGC** (**mg/g extract)**	not include in model	7.53 ± 0.77	–	5.83 ± 0.01^b^
**EGCG** (**mg/g extract)**	32.723	37.78 ± 0.58^a^	15.45	17.97 ± 0.54^b^
**TPC (mg GAE/g)**	285.137	253.38 ± 7.88^a^	11.14	199.03 ± 11.64^b^
**DPPH (% inhibition)**	50.848	45.45 ± 2.03^a^	11.89	29.71 ± 1.20^b^
**ABTS (mol Trolox/g)**	43.582	40.17 ± 0.34^a^	7.83	30.24 ± 0.55^b^
**FRAP (mmol FeSO**_**4**_**/g)**	29.248	27.06 ± 0.25^a^	7.50	22.38 ± 0.26^b^
**sirtuin 1 enzyme-stimulating activity (FIR)**	20.205	22.63 ± 0.17^a^	10.71	9.37 ± 0.60^b^

Note: ^1^The response variables were calculated using the predicted equations, with the optimal conditions of the independent variables set as follows: an intensity of electric field of 5 kV, number of pulses of 3000 pulse and ratio of green tea to water of 0.14 g/mL.

^2^The extraction process involved the use of distilled water, an extraction time of 6 h, an extraction temperature of 25 °C and a ratio of green tea to water of 0.14 g/mL.

3Means of three experimental replicates (mean ± SD).

4%Error = % prediction error = [(Observed values - Predicted value)/Predicted value] x 100 %.

a-b Values within the same row that have different superscript letters were significantly different from each other at a significance level of P < 0.05.

When comparing the optimal values of the response variables obtained from the predicted model, they were found to be higher than the values of the response variables obtained from water extraction. The results of the study demonstrated that the concentrations of catechin and its derivatives extracted using PEF under optimized conditions were significantly greater than those obtained through water extraction (non-PEF). Specifically, the levels of C, EC, ECG, EGC and EGCG were found to be approximately 36.51 %, 15.72 %, 31.20 %, 22.58 % and 52.44 % higher in the PEF-extracted samples, compared to the non-PEF-extracted samples. The findings of this study were consistent with the research which reported an increase in total catechin content by 15.43 % for PEF and 25.09 % for intense pulsed light compared to untreated leaves during pretreatment [54]. Their study confirmed that both PEF and intense pulsed light had a beneficial impact on enhancing the extraction of tea catechins from green tea leaves using subcritical water extraction. The TPC of the green tea extract obtained from PEF extraction was found to be approximately 21.34 % significantly higher compared to the extract obtained from water extraction. The increased catechin, catechin derivative content and phenolic compounds of the green tea extract obtained from PEF extraction can be attributed to the effect of the electric field. The electric field causes the tissue surface to extend and act as a conductor, resulting in the accumulation of charge on both sides. This accumulation leads to the generation of a high local electric field and subsequent local breakdown, which releases sufficient energy to create punctures or holes [55]. Furthermore, the penetration of plant tissue barriers applying short high-voltage pulses could be explored more thoroughly through electro-permeabilization processes. The disintegration index (Zp) can used to indicate the level of cell membrane electro-permeabilization in plant tissue for considering the electric field strength [56]. Base on this experiment, green tea powder was soaked in water in a chamber for around 5 min to increase the electro-permeability of green tea tissue by wet form, assisting in the extraction process using PEF [56,57]. The antioxidant activities, as measured by DPPH, ABTS and FRAP assays, were found to be higher in the extract obtained from PEF extraction compared to the non-PEF extraction. This increase in antioxidant activities can be attributed to the enhanced levels of catechin, catechin derivatives and phenolic compounds in the PEF-extracted sample. The DPPH, ABTS and FRAP values of the green tea extract obtained from PEF extraction were observed to be significantly higher, with increases of approximately 35.82 %, 24.72 % and 17.29 %, respectively, compared to the extract obtained from water extraction. It is widely recognized that catechins present in green tea exhibit highly effective DPPH radical scavenging activity, ABTS-scavenging capacity and play a significant role in the stoichiometry of Fe^3+^ reduction [58,59]. Additionally, the green tea extract obtained from PEF extraction exhibited a significantly higher sirtuin 1-activity, with an increase of approximately 58.59 %, compared to the extract obtained from water extraction. The activity of the sirtuin 1 enzyme was found to be enhanced when green tea was subjected to pretreatment with PEF before water extraction, related to an increase in the content of EGCG. The results of this study align with the finding demonstrated that EGCG activates autophagic pathways by inducing the sirtuin 1-activity [60].

### 3.4 Surface morphology and FTIR analysis during PEF extraction

**Fig. 2:**
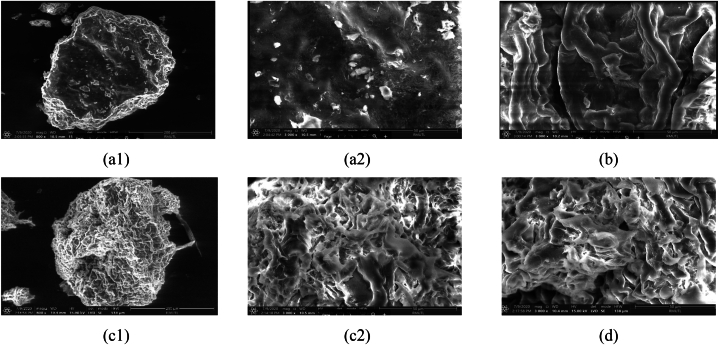
Surface morphology of green tea powder (a1-a2) before extraction in 800x and 3,000x (b) water extraction (non-PEF) in 3,000x (c1-c2) pretreatment by PEF in in 800x and 3,000x and (d) water extraction after PEF in 3,000x

The surface morphology of green tea powder before extraction, as well as the effects of pretreatment with PEF and non-PEF conditions, were examined using scanning electron microscopy. Fig. 2 illustrates the results of this analysis. The surface of untreated green tea powder exhibited a relatively smooth texture, with the presence of dispersed regular pellets on the cell surface. Notably, no clearly visible pores were observed, as depicted in Fig. 2(a1-a2). In the case of non-PEF or water extraction in Fig. 2(b), the surface of the green tea powder exhibited relatively large fibrous swelling. This swelling may be attributed to the diffusion of water during the extraction process. Subsequently, the water penetrated the green tea powder and extracted water-soluble compounds such as catechins, caffeine, chlorophyll, organic acids and vitamins [61,62]. Fig. 2(c1-c2) illustrates the destruction of fibrous swelling, resulting in a torn fiber appearance and the emergence of small pores. This phenomenon can be attributed from the accumulation of charges on the surface, which causes the reorientation of electrical charges or dipoles within the structure [41,55].

As a result, the enhanced leaching of the target compounds in green tea resulted in an increased content of catechins, total phenolic compounds, antioxidant activities and sirtuin 1 enzyme-stimulating activity, as previously mentioned. Following PEF treatment and subsequent water extraction, the torn fibrous appearance did not show significant differences compared to after PEF treatment. However, there was an observed increase in fibrous swelling due to the diffusion of water into the green tea powder. These surface characteristics are depicted in Fig. 2(d).

**Fig. 3:**
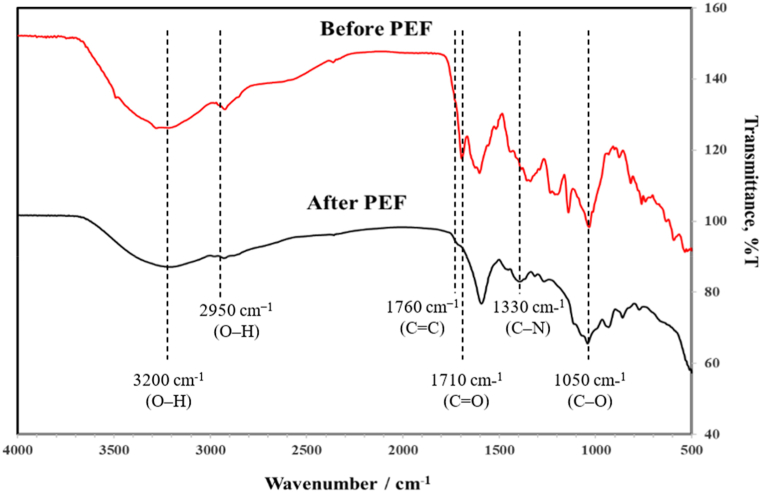
The FTIR spectra of green tea powder before and after PEF extraction.

Furthermore, the impact of PEF on the physical properties of green tea powder during extraction can also be elucidated through FTIR analysis. Fig. 3 displays the FT-IR spectra of green tea powder before and after extraction using PEF. The transmittance (%T) of the functional groups of the active components in green tea powder were identified using red and black lines, representing before and after PEF extraction.

The vibrational properties of catechin and its derivatives, which constitute approximately 13–30 % of the dry weight of green tea [63], showed variations of before and after undergoing PEF treatment. The hydroxy groups in both the before and after PEF samples exhibited peak vibrations at 3400 - 3100 cm^−1^ (O–H) indicating the presence of phenolic rings [64,65]. Both un-galloylated catechins (EC and EGC) and galloylated catechins (ECG and EGCG) are characterized by the presence of multiple hydroxyl groups [66]. The spectral data obtained from the extracted green tea showed decreased peak intensities, which can be attributed to the diminished concentration of hydroxyl groups in catechin and its derivatives because of PEF extraction. Furthermore, a significant reduction in the vibrational bands associated with the stretching of amino acid at 1050 cm^−1^ (C–O) [67] was observed after PEF treatment. This vibrational band can be attributed to the presence of l-Theanine, which is a unique and distinctive amino acid found in green tea [49,50]. The vibrational bands at 1710 cm^−1^ and 1760 cm^−1^ were attributed to the C=O stretch in polyphenols and the C=C stretch in aromatic ring, respectively [65,67]. The weak bands observed at 1330 and 2950 cm^−1^ can be attributed to the stretch of C–N in amide-I in protein and stretch of the O–H in carboxylic acid [65,67]. The decrease in vibrational bands associated with hydroxyl groups, aromatic rings, amino acids, proteins and carboxylic acids indicates the extraction of catechin and its derivatives, as well as other compounds, from green tea powder after treatment by PEF.

strong band at 1627 cm^−1^ is attributed to the C=C

stretch in aromatic ring and C=O stretch in polyphenols.

The results suggest that the application of an electric field pulse affects the structural arrangement of green tea, leading to an increased release of catechin and its derivatives into the water. Consequently, the surface of green tea can be disrupted through the application of a pulse electric field, resulting in a higher content of catechin and its derivatives. Based on the findings from SEM and FT-IR analysis, it can be concluded that the application of PEF improves the extraction efficiency of green tea by altering its structural arrangement and enhancing the diffusion of targeted components on the surface of green tea.

## 4 Conclusion

This study utilized the PEF technique to enhance the extraction of bioactive compounds from green tea. The results demonstrated that the optimization of PEF parameters, including an electric field intensity of 5 kV/cm, 3000 pulses, and a green tea powder to water ratio of 0.14 g/mL, using the response surface methodology with a central composite design, effectively predicted the impact of these variables on the content of catechin and its derivatives, antioxidant activities and sirtuin 1-activity. The results of the study demonstrated a significant enhancement in the extraction yield of catechins (C, EC, ECG and EGCG), antioxidant activities (DPPH, ABTS and FRAP) and sirtuin 1-activity from green tea with an increase in the number of pulses. In particular, the PEF-treated green tea extract exhibited a higher amount of catechin, and its derivatives compared to the extract obtained without pretreatment. This can be attributed to the enhanced mass transfer facilitated by PEF, which promotes the release of bioactive components from the surface of green tea. In conclusion, this study demonstrated that PEF is a promising technique for enhancing water extraction from green tea, which can contribute to responsible consumption and production for health-conscious consumers. Furthermore, these findings have led to innovations in the plant extraction industry and infrastructure, particularly in terms of developing green and economically efficient processes.

## Data availability statement

All data generated or analyzed during this study are included in this article.

## CRediT authorship contribution statement

**Nuttinee Salee:** Writing – original draft, Methodology, Investigation, Conceptualization. **Srisuwan Naruenartwongsakul:** Visualization, Supervision, Conceptualization. **Wantida Chaiyana:** Validation, Software, Resources. **Artit Yawootti:** Resources, Methodology. **Kanyarat Suthapakti:** Writing – review & editing, Validation, Conceptualization. **Piyawan Simapaisarn:** Writing – review & editing, Validation. **Worrapob Chaisan:** Writing – review & editing. **Niramon Utama-ang:** Writing – review & editing, Visualization, Validation, Supervision, Conceptualization.

## Declaration of competing interest

The authors declare that they have no known competing financial interests or personal relationships that could have appeared to influence the work reported in this paper.
